# Recognition of emotions expressed on the face impairments in Parkinson’s disease

**Published:** 2020-01-05

**Authors:** Kaveh Shafiei, Mohammad Ali Shafa, Forugh Mohammadi, Ali Arabpour

**Affiliations:** 1 Neurology Research Center, Kerman University of Medical Sciences, Kerman Iran; 2 Department of Neurology, School of Medicine, Kerman University of Medical Sciences, Kerman, Iran

**Keywords:** Language Delay, Age of Onset, Children, Iran, Epilepsy

## Abstract

**Background:** Facial emotion recognition (FER) is a complex process, involving many brain circuits, including the basal ganglia that its motor involvement causes Parkinson's disease (PD). The previous studies used different tools for assessment of FER in PD. There is a discrepancy between the results of these studies due to different tools. In this study, we used a modified version of the Multimodal Emotion Recognition Test (MERT) to compare patients with PD to healthy controls (HCs).

**Methods:** It was a cross-sectional study with primary objective of the mean percentage of the correct answers in MERT. Subjects had to name the emotions presented with different modalities.

**Results: **30 subjects were recruited and assessed in each group. The mean total MERT score was significantly lower in subjects with PD compared to HCs (35.0% vs. 44.5%). FER was significantly better when emotions were presented by video and worse when presented by still pictures. Both subjects with PD and HCs had lower MERT scores in recognizing negative emotions. There was no significant correlation between the duration and severity of PD and MERT score.

**Conclusion:** Our study provided more pieces of evidence for impairment of FER in PD for recognizing emotions like sadness, disgust, and fear compared to happy expressions.

## Introduction

When James Parkinson was writing "the essay on shaking palsy" in 1817, he was clearly describing that expressive face would become masked. On the flip side, this raises the question that: Does reduced facial emotion expression (FER) in Parkinson's disease (PD) lead to impairment in emotion recognition?

The impairment of FER in PD has been investigated in many fantastic studies, but with different and sometimes contradictory results.^1^^-^^6^ The FER represents a prominent non-motor symptom.^7^ Recent functional and electrophysiological studies have demonstrated that FER is not related to a single neuronal network.^8^^,^^9^ We already know that many of these circuits are interrupted which results in motor and non-motor symptoms of PD.^10^

Majority of studies have shown that FER is impaired even at early stages of PD and deteriorates as the disease progresses.^11^ There are also studies that have not found any impairment neither in FER nor in prosody.^1^^,^^12^

The Multimodal Emotion Recognition Test (MERT) which has developed by the Geneva Emotion Recognition Group, is a dynamic and multimodal actor portrayal, containing a large number of emotions and is based on modern psychometric principles [item response theory (IRT)]. It has been shown that MERT fits the emotion subtests and its overall measurement precision is satisfactory. It is a promising instrument to measure emotion recognition in a more ecologically valid and comprehensive fashion than the previous tests.

We hypothesized that the discrepancy between these studies was caused by using different tools to measure the ability of patients with PD in FER. So, we decided to compare patients with PD to healthy controls (HCs) with a multimodal emotional recognition assessment tool for FER.

## Materials and Methods

It was a cross-sectional study with the primary objective to compare the MERT mean score between patients with PD and matched HCs.

We selected patients with PD who came to our neurology outpatient clinic at Shafa University Hospital, Kerman, Iran. Our inclusion criteria were: no hearing or visual problem, diagnosis of idiopathic PD according to UK Brain Bank criteria, stable clinical condition and no changes in medication at least four weeks before assessment, no significant cognitive deficits [Mini-Mental State Exam (MMSE) score > 24], no affective disorder based on Diagnostic and Statistical Manual of Mental Disorders, Fourth Edition, Text Revision (DSM-IV-TR) criteria [Beck Depression Inventory (BDI) score < 10], and no other neurological disorders.

MERT consists of 30 video clips of actors for each of 10 emotions (despair, cold and hot anger, anxiety, panic fear, happiness, elated joy, disgust, contempt, and sadness). The emotions presented to the subjects in four modalities: Still picture, Videos, Videos with audio and only audio, and in total of 120 items. The auditory content of the test was a standard pseudo-linguistic sentence (i.e., a sentence without meaning). Subjects were asked to sit in front of a monitor and identify the presented emotions.

Our team had to modify the original version of MERT for three reasons: 1) the MERT is an online web-based test (which most of the time makes it impossible in our clinical setting), 2) the software works on Java platform which was not available in Iran due to United States (US) sanctions, and 3) we were looking for not only the total mean score but also the score for each separate emotion. So, we had to rebuild our refined version of the MERT. Our version was offline and gave us not only the final score but also the score for the different subtype of emotions. Each session lasted nearly 45 minutes.

To be sure that our subjects understand their task, they had to describe an example of a scenario for each emotion (for example: "show me on your face when you are happy") before each session.

Following background data were also collected: demographic and clinical information [disease duration, levodopa equivalent daily dose (LEDD)] and Hoehn and Yahr score.

The MERT mean score was tested for normality assumption using the Shapiro-Wilk test. Two-way analysis of variance (ANOVA) was applied to show the normal distribution.

We would consider the mean value in each group for each ranked emotion for both recognition and expression. A P-value < 0.05 was considered significant.

## Results

We recruited 30 patients with PD and the same number of HCs. As shown in table 1, both groups had no significant differences in sex distribution, age, or MMSE. The mean modified Hoehn and Yahr score was 2.3 for disease severity which means that patients with PD had mild bilateral disease severity.

**Table 1:** Demographic findings in patients with Parkinson's disease (PD) and healthy controls (HCs)

**Variable**	**Patients ** **with PD**	**Normal ** **controls**	**P**
Female/male (n)	13/17	16/14	0.54
Age (year)	58.2 ± 7.0	57.1 ± 5.0	0.76
MMSE	28.1 ± 3.0	28.3 ± 4.0	0.40
Hoehn and Yahr score	2.3 ± 0.4	NA	
43-48	16 (20.5)	16 (20.5)	
49-60	16 (20.5)	16 (20.5)	
Total	78 (100)	78 (100)	

Data are presented as mean ± standard deviation (SD) or number and percentage, NA: Not applicable

**Figure 1:**
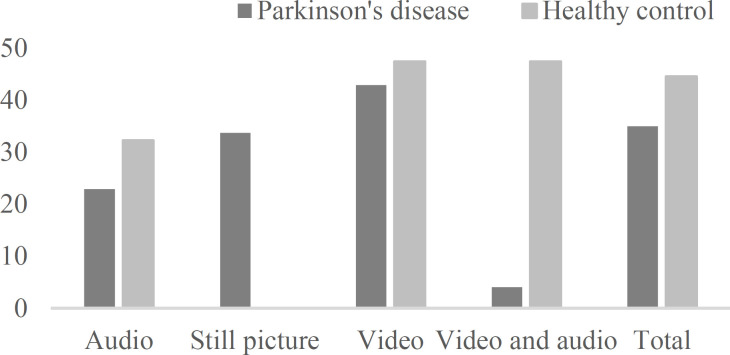
Mean Multimodal Emotion Recognition Test (MERT) score in patients with Parkinson’s disease (PD) and healthy controls (HCs) based on different modality of emotion presentation

Mean total MERT score of patients with PD was significantly lower than HCs (35.0% vs. 44.5%), regardless of modality in which emotion was presented (Figure 1). FER in both groups was significantly better when emotions were presented by video and worse when presented by still pictures.

**Figure 2:**
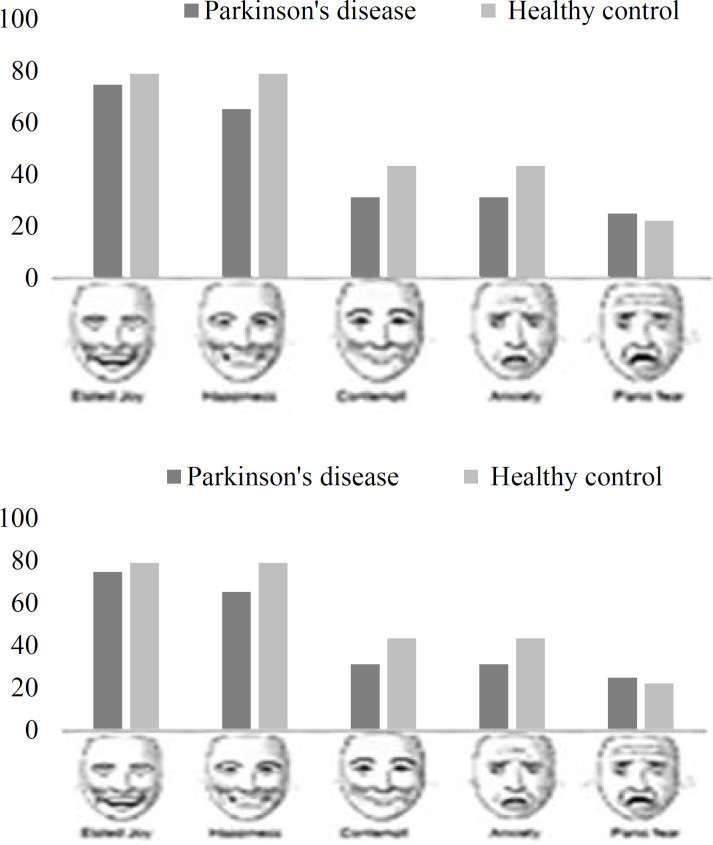
The Multimodal Emotion Recognition Test (MERT) score (percentage of correct answers) in patients with Parkinson's disease (PD) and healthy controls (HCs) based on different emotions. Arrows show statistically significant differences.

As shown in figure 2, patients with PD had significantly lower score in recognizing contempt (31.7% vs. 44.2%), anxiety (29.3% vs. 44.3%), disgust (75.8% vs. 78.6%), cold anger (68.8% vs. 77.9%), and hot anger (31.7% vs. 44.2%) (P < 0.05).

Further statistical analysis by Pearson correlation coefficient showed that there was no significant correlation between the duration and severity of PD and MERT score.

## Discussion

This study showed that there was FER impairment in PD. This impairment is more prominent for negative and dynamic emotions than positive and still ones. Although changes in FER in PD has been studied in the past few years,^13^ as far as our knowledge, our study is novel in using the MERT with different modalities for presenting emotions.

The review article by Argaud et al. published in the Journal of Movement Disorders in 2018, has given the most in-depth insight on that issue.^4^ This review article showed that there was an actual deficit in FER in patients with PD. By disease progression, not only negative emotions but also positive ones have been affected.^14^ A few reasons might explain why negative emotions are affected early. First, the recognition sensitivity of anger or happiness is higher than surprise, just because of more facial muscles’ contract in expressing them.^15^^,^^16^ Second, different neuronal pathways exist for positive and negative emotion recognition processing.^17^ The negative emotion is preattentive and this leads to an autonomic reaction to stimuli that are threatening to subject, like fear or anger. These fast pathways encompass the pulvinar, amygdala and striatum and make the fast visual information processing mechanism.^18^ Some investigators called this "angry faces advantage".^19^^,^^20^

Early structural imaging studies also revealed a positive correlation between following brain areas and specific FER: 1) sadness: the right orbitofrontal cortex (OFC), amygdala, and postcentral gyrus, 2) anger: the right occipital fusiform gyrus, ventral striatum, and subgenual cortex, and 3) disgust: the anterior cingulate cortex (ACC).^18^ The predominant right-hemispheric affection in left-onset PD is associated with more impairment in FER.^17^ So, patients with PD probably not only misread emotion expression in others but also in themselves as well.

The deficit in FER has a negative impact on interpersonal relationship in PD.^21^ However, the debate on this subject is still alive, mostly due to different tools for assessing FER.^13^

## Conclusion

Our study provides more pieces of evidence to consider the impairment of FER in PD as non-motor symptoms. Patients with PD have more impairment in recognizing negative emotions like sadness, disgust, and fear than happy expressions. Recognizing other emotions, conveyed by either face or voice, is also affected, as the overlapping mechanism of the emotional circuitry. FER is better when presented by videos than still pictures.

## Notes:

**How to cite this article:** Shafiei K, Shafa MA, Mohammadi F, Arabpour A. Recognition of emotions expressed on the face impairments in Parkinson’s disease. Curr J Neurol 2020; 19(1): 32-5.
